# Physiological serum 25-hydroxyvitamin D concentrations are associated with improved thyroid function—observations from a community-based program

**DOI:** 10.1007/s12020-017-1450-y

**Published:** 2017-10-24

**Authors:** Naghmeh Mirhosseini, Ludovic Brunel, Giovanna Muscogiuri, Samantha Kimball

**Affiliations:** 1 Pure North S’Energy Foundation, 326 11th Avenue SW, Suite 800, Calgary, AB T2R 0C5 Canada; 2 Naturmend Integrative Medical Clinic, 905 1st Ave NE, Calgary, AB T2E 2L3 Canada; 3 IOS and Coleman Medicina Futura Medical Center, via Alcide De Gasperi 107/109/111, 80011 Acerra (Napoli), Italy; 4 St. Mary’s University, 14500 Bannister Road, Calgary, AB T2X1Z4 Canada

**Keywords:** Thyroid function, Vitamin D, 25-Hydroxyvitamin D, Autoimmune thyroid, Anti-thyroid antibodies, Hypothyroidism

## Abstract

**Purpose:**

Vitamin D deficiency has been associated with an increased risk of hypothyroidism and autoimmune thyroid disease. Our aim was to investigate the influence of vitamin D supplementation on thyroid function and anti-thyroid antibody levels.

**Methods:**

We constructed a database that included 11,017 participants in a health and wellness program that provided vitamin D supplementation to target physiological serum 25-hydroxyvitmain D [25(OH)D] concentrations (>100 nmol/L). Participant measures were compared between entry to the program (baseline) and follow-up (12 ± 3 months later) using an intent-to-treat analysis. Further, a nested case-control design was utilized to examine differences in thyroid function over 1 year in hypothyroid individuals and euthyroid controls.

**Results:**

More than 72% of participants achieved serum 25(OH)D concentrations >100 nmol/L at follow-up, with 20% above 125 nmol/L. Hypothyroidism was detected in 2% (23% including subclinical hypothyroidism) of participants at baseline and 0.4% (or 6% with subclinical) at follow-up. Serum 25(OH)D concentrations ≥125 nmol/L were associated with a 30% reduced risk of hypothyroidism and a 32% reduced risk of elevated anti-thyroid antibodies. Hypothyroid cases were found to have higher mean serum 25(OH)D concentrations at follow-up, which was a significant positive predictor of improved thyroid function.

**Conclusion:**

The results of the current study suggest that optimal thyroid function might require serum 25(OH)D concentrations above 125 nmol/L. Vitamin D supplementation may offer a safe and economical approach to improve thyroid function and may provide protection from developing thyroid disease.

## Background

Most tissues in the body have vitamin D receptors and thousands of genes are responsive to active vitamin D, 1-25-dihydroxyvitamin D [1,25(OH)_2_D], suggesting a role for vitamin D in the normal physiological function of most organ systems, including the thyroid. The thyroid is activated through the hypothalamus-pituitary-thyroid axis which is remarkably prone to circadian and seasonal changes [1]. There is seasonal variability to serum thyroid-stimulating hormone (TSH) concentrations with the highest levels in autumn-winter and the lowest in spring-summer [2–4]. Vitamin D levels are also affected by seasonal variability and serum 25 hydroxyvitamin D [25(OH)D) levels closely correlate with sun exposure and seasonality, with more vitamin D deficiency (<50 nmol/L) prevalent during the colder seasons [5, 6].

Evidence is increasingly indicating low vitamin D status as a risk factor for autoimmune disease, particularly multiple sclerosis, and including thyroid disease [7–11]. Moreover, TSH levels are closely associated with vitamin D status. During the winter months when vitamin D production is negligible and levels are at a nadir for instance, thyroid cells are less responsive to TSH and, as a result, thyroid hormones (T4) decrease and serum TSH levels increase [4, 12]. Vitamin D supplementation, targeted at achieving and maintaining serum 25(OH)D levels above 100 nmol/L, may preserve normal human physiology, decrease the risk of autoimmunity and improve immune function in autoimmune disorders [13–16].

Many thyroid disorders have an autoimmune etiology, characterized by a loss of immune system homeostasis [17]. Given the immunomodulatory and anti-inflammatory roles of vitamin D, supplementation may act to suppress autoimmune activity in thyroid disease and improve thyroid function. A recent meta-analysis including 20 case–control studies found that serum 25(OH)D was lower in individuals with autoimmune thyroid disease (AITD) compared with healthy controls (OR = 2.99, 95% CI 1.88–4.74) and that AITD was more likely to develop with low serum 25(OH)D [18]. Vitamin D deficiency is a common feature in thyroid disorders [19] and low serum 25-hydroxyvitamin D [25(OH)D] concentrations are associated with the development of both Hashimoto’s thyroiditis and Grave’s disease [20, 21]. The onset and progression of thyroid cancer has been linked with impaired signaling of 1,25(OH)_2_D through the vitamin D receptor and lower 25(OH)D concentrations were associated with more severe hypothyroidism [22]. Correcting serum 25(OH)D status appears to improve thyroid function by reducing circulating thyroid-stimulating hormone (TSH) [23, 24].

Thyroid autoimmunity, presenting with increased thyroid autoantibody levels, anti-thyroid peroxidase (anti-TPO) anti-thyroglobulin (anti-TG) antibodies, is associated with vitamin D deficiency [serum 25(OH)*D* <50 nmol/L] [19, 25, 26]. Despite the scarcity of clinical trials investigating vitamin D supplementation effects on thyroid function, the available studies collectively suggest clinical benefit from vitamin D supplementation in the treatment of autoimmune thyroid disorders with reductions in anti-thyroglobulin (anti-TG) and anti-thyroid peroxidase (anti-TPO) antibody levels [27–31].

In Canada, one in ten suffer from a thyroid disorder, half of them undiagnosed [32]. Overall, a third of Canadians are vitamin D deficient [25(OH)*D* <50 nmol/L] and less than 10% have levels above 100 nmol/L [33]. Vitamin D may be an easily modifiable risk factor for autoimmune thyroid disease and supplementation may be used as an adjuvant for treatment [34]. The present analysis utilized a large database of participants in a wellness program receiving vitamin D supplementation, with average doses of 6000 IU/d. We investigated the association between 25(OH)D status and thyroid function before and after treatment. We further examine differences between hypothyroid and euthyroid patients.

## Methods

### Study design and population

This database analysis is a secondary use of data collected as part of the standard of care for participants in a health and wellness program provided by the Pure North S’Energy Foundation (Pure North), a not-for-profit organization in Calgary, Alberta, Canada. In the Pure North program, participant visits occur approximately yearly and include gathering medical history, consultation and lifestyle recommendations by a health care professional (medical doctor, naturopathic doctor, or nurse practitioner), blood work and anthropometric measurements. A dataset was constructed to include all participant data from January 1st 2010 to December 31th, 2016 who had consented to the use of their anonymized data for research and who met the inclusion criteria. To be included in the dataset participants had to have a program entry measurement for all of the following: 25(OH)D, free T3 (FT3), and T4 (FT4), thyroid stimulating hormone (TSH), anti-TPO, anti-TG, and high-sensitivity C reactive protein (hs-CRP). In addition, the following information was included if it was available: ethnicity, gender, body mass index (BMI), season of the observation (November–April was considered winter and May–October as summer), medical history of thyroid disorders and medications, vitamin D supplementation intake and thyroid symptom measures (described below). To characterize the association between serum 25(OH)D and thyroid function, comparisons were made at baseline and between baseline and follow-up using intent-to-treat analyzes.

Secondly, we utilized a nested case–control design, in which hypothyroid participants (cases, *n* = 103) were matched in a 1:4 ratio to control participants (*n* = 412) based on age, sex, BMI and the first two digits of their postal code (to geographically account for some socioeconomic factors). In this investigation, we examined the effect of serum 25(OH)D longitudinally on thyroid function. This study was approved by the Research Ethics Board at St. Mary’s University, Calgary (File # 007FA2017).

### Thyroid measures

Participants were interviewed by a health care practitioner to collect medical history, medication use (iodine, desiccated thyroid or armor thyroid, synthroid or levothyroxine, or any other thyroid medications). To assess suboptimal thyroid function a series of questions were asked during the consultation to evaluate the most common symptoms of hypothyroidism: brain fog, macroglossia, low mood, unrefreshing sleep, cool body temperature, weight gain, and low energy level. Blood work assessed thyroid function measures.

### Pure north program and vitamin D supplementation

The goal of the Pure North program is to optimize health and prevent chronic disease. The Pure North program provides education, lifestyle advice, and nutritional supplements to meet individual requirements. The goal of the program is to achieve optimal nutritional intake with a focus on optimizing vitamin D status, defined as serum 25(OH)D concentrations ≥100 nmol/L. Vitamin D3 supplementation is individualized to target an optimal 25(OH)D and doses of vitamin D3 are often in excess of the UL (4000 IU/d) given under medical supervision. The data collected as part of this program provided a unique opportunity to investigate the role of a wide range of 25(OH)D concentrations on thyroid function and autoimmunity.

### Laboratory assessments

Sample preparation and biochemical measurements were performed mostly by Doctor’s Data Laboratory, Chicago [DD], a fully accredited laboratory by Clinical Laboratory Improvement Amendments (CLIA). On some occasions, biomarker results were obtained from other certified laboratories (Calgary Laboratory Services, Meridian Valley Lab). All laboratory testing was validated according to ongoing externally provided accreditation test samples.

Serum 25(OH)D was measured using liquid chromatography and tandem mass spectrometry (LC/MS-MS), with an assay CV of 2.4%. Thyroid function parameters including serum free triiodothyronine (FT3; reference range: 2.5–5.7 pmol/L), free thyroxine (FT4; reference range: 7.7–20.6 pmol/L), Thyroid Stimulating Hormone (TSH; reference range: 0.45–3 mU/L), Thyroglobulin (TG; reference range: M: <50 µg/L, *F*: <30 µg/L), anti-peroxidase antibody (anti-TPO; reference range: < 9 kIU/L) and anti-Thyroglobulin antibody (anti-TG; reference range: < 4 kIU/L), were measured on a Beckman Coulter automated analyzer, using chemiluminescent immunoassays. Inter-assay CV was 5% for TSH, 8.3% for FT3, 3.6% for FT4, 6.9% for anti-TPO antibody and 6.6% for anti-TG antibody. High-sensitivity C reactive protein (hs-CRP; reference range: <1.0 mg/L) was measured using the immunoturbidimetric method with an inter-assay CV of 2.5%.

### Participant subgroups

Vitamin D deficiency was defined as serum 25(OH)D concentrations<50 nmol/L [35] and optimal concentrations ≥100 nmol/L [16]. Subclinical hypothyroidism was defined as serum TSH concentrations >3 mlU/L, with serum concentrations of FT4 and FT3 within their respective references ranges. Hypothyroidism was defined as serum TSH > 3 mlU/L with serum FT4 < 10.3 pmol/L and serum FT3 either within the reference range or < 2.57 pmol/L.

Several patients started thyroid replacement hormones as a results of the testing conducted by Pure North. Participants with undiagnosed or poorly managed hypothyroidism were referred to their family physician. In some cases patients declined referral or follow up, refused treatment, did not comply with medication, or their physician did not believe that replacement hormones were needed at that time. Any change in medication, especially when close monitoring is required to reach an appropriate dose, was done outside of the Pure North program via a primary care practitioner.

There has been some debate on the correct upper limit of the reference range for TSH concentrations in euthyroid subjects [36–38]. Here we follow the 2002 recommendations of the American Association of Clinical Endocrinologists, we used the upper limit of the serum TSH euthyroid reference range of 3 mlU/L, which represents the 95% of normal euthyroid population [39]. Also, concentrations above this threshold increase the odds ratio of developing hypothyroidism over the 20 years, especially if thyroid antibodies were elevated [40]. Thyroid autoimmunity was defined as a serum level of anti-TPO ≥ 9 klU/L and/or anti-TG was ≥ 4 klU/L [32].

### Statistical analysis

Data were analyzed using SPSS version 23 (SPSS Inc., Chicago, IL). Descriptive analyzes were performed to show the distribution of categorical data. Intent-to-treat analyzes was used to compare measures between baseline and follow-up. The results of *per-protocol* analysis are available upon request. The follow-up average for each biomarker was inserted rather than a missing value for those participants who had the baseline value. Paired samples t-tests were performed to evaluate changes in thyroid function measures and other metabolic parameters over time. Independent samples t-tests were utilized to compare mean changes according to compliance groups. Chi-square tests were performed to determine the association between reported thyroid assessment parameters and serum 25(OH)D status and vitamin D supplementation dose. Relative Risks (RR) were calculated. Univariate analyzes were used to compare changes in thyroid markers between cases and controls with respect to serum 25(OH)D levels. Binary logistic regressions were performed to look at the association between vitamin D and B12 status with respect to thyroid function measures and to investigate the effect of vitamin D and/or vitamin B12 status on changes in thyroid function over time, considering probable confounding parameters including age, sex, BMI, season of observation, thyroid medication or thyroid-related supplementation. Because serum TSH, anti-TPO, anti-TG and TG levels are higher than the reference range in hypothyroidism and thyroid autoimmune disorders, improvement was defined as decreased levels over time in regression models. In contrast, serum FT3 and FT4 are lower than normal and improvement was defined as an increase in levels. Significance was defined as *p* < 0.05.

## Results

### Pure north population

#### Baseline demographics

Baseline demographics are presented in Table 1. Mean age was 48 ± 16 years with 58% female (*n* = 11,017). Vitamin D supplement use was reported by 43% of participants at baseline, greater than the estimates for Canadian of more than 32% [33]. The BMI distribution was 35.3% normal (18.5–24.9 kg/m^2^), 36.1% overweight (25–29.9) and 28.6% obese (≥30) which was in agreement with Canadian population averages [41]. Mean baseline serum 25(OH)D concentrations were 78 ± 34 nmol/L with 19% vitamin D deficient [<50 nmol/L], 80% below the target (<100 nmol/L) and 92% < 125 nmol/L. Serum 25(OH)D level was significantly lower during the winter season (61 ± 28 nmol/L) compared to the summer season (70 ± 26 nmol/L) in participants who did not take vitamin D supplements at program entry. Vitamin D deficiency was seen in 37.5% of these participants in winter and 23% in summer.

**Table 1:** Baseline demographics

Parameter	N	Percentage (%)
Age, years	11,017	48 ± 16 (18–95 years)
Gender	11,017	
Female	6378	58
Male	4649	42
Body Mass Index, kg/m^**2**^	10,554	27.6 ± 5.7
Normal weight (18.5–24.99)	3730	35.3
Overweight (25–29.99)	3807	36.1
Obese (≥30)	3017	28.6
Medication history	4411	
Desiccated thyroid (Armor thyroid)	81	1.6
Synthroid	592	13.4
Other thyroid medications	100	2.3
Supplementation history		
Iodine	1788/11,017	14.8
Magnesium	446/8926	4.9
Niacin	56/8779	0.6
Vitamin D	4694/11,016	42.6
Thyroid Assessment Questionnaire		
Brain fog	3761/10,176	37.0
Low energy level	3643/6865	53.1
Macroglossia	1343/9810	13.7
Low mood	3534/10,147	34.8
Unrefreshing sleep	4993/10,329	48.3
Cool body temperature	3124/10,097	30.9
Weight gain	2715/10,004	27.1
Serum 25(OH)D status, nmol/L	11,017	
<50	2101	19.1
50–100	6660	60.4
100–150	1867	16.9
150–200	289	2.6
200–250	77	0.7
≥250	23	0.2

Age and BMI presented as Mean±SD

Participants who were vitamin D deficient and did not take any vitamin D supplement at program entry had higher serum TSH in winter (2.54 ± 2.6 mU/L) rather than summer (2.40 ± 2.3 mU/L) (*p* = 0.1). Meanwhile, serum FT4 was significantly lower in winter (14.2 ± 2.8 pmol/L) compared to summer (14.8 ± 2.9 pmol/L) (*p* < 0.001). We found a negative correlation between serum TSH and 25 (OH)D levels [Pearson *r* = −0.04, *p* = 0.01], indicating that decreased levels of serum 25(OH)D in winter were correlated with increased levels of serum TSH.

#### Comparison between baseline and follow-up

We performed a comparison between baseline and follow-up (12 ± 3 mo) for 11,017 participants, using Intent-To-Treat analysis. Demographics did not show any significant changes over time, except higher consumption of iodine, magnesium and vitamin D at follow-up, as well as significant improvement in thyroid symptoms like low energy level, macroglossia, unrefreshing sleep and weight gain (Supplementary Table 1). All thyroid measures differed statistically between baseline and follow-up yet mean values for FT3, FT4, TG and TSH remained within their respective reference ranges ( ≤ 9kU/L for anti-TPO, ≤ 4kU/L for anti-TG) (Table 2). There was a weak but significant negative correlation between TSH and thyroid hormones (FT3 and FT4) at both baseline (*r* = −0.08 for FT4, *r* = −0.07 for FT3) and follow-up (*r* = −0.07 for FT4 and *r* = −0.10 for FT3). Also, changes in thyroid hormones over time were negatively correlated with changes in TSH level (*p* < 0.001). Mean anti-thyroid antibody levels were above their respective reference ranges and were found to be significantly lower at follow-up, with a mean change in anti-TPO of −9.8 ± 65 kU/L and anti-TG of −25.4 ± 70 kU/L. For those who had elevated anti-thyroid antibody levels, at follow-up 77.5% were within the reference range for anti-TG and 42.2% for anti-TPO.

**Table 2:** Comparison of measures between baseline and one-year follow-up

Parameter	N	Baseline (mean ± SD)	Follow-up (MEAN ± SD)	P-value
FT3 (pmol/L)	11,017	4.9 ± 0.8	4.6 ± 0.2*	<0.001
FT4 (pmol/L)	11,017	14.8 ± 2.9	13.5 ± 0.9*	<0.001
Anti-TPO (kU/L)	11,017	32.2 ± 92.0	22.4 ± 41.0*	<0.001
Anti-TG (kU/L)	11,017	40.1 ± 112	14.7 ± 28.7*	<0.001
TSH (mU/L)	110,17	2.53 ± 2.1	2.13 ± 0.8*	<0.001
TG (µg/L)	6509	29.2 ± 27.0	25.1 ± 5.0*	<0.001
hs-CRP (mg/L)	10,968	2.71 ± 4.6	2.40 ± 2.2*	0.04
25(OH)D (nmol/L)	11,017	78 ± 34	110 ± 22*	<0.001
Vitamin D dose (IU/d)	11,017	1436 ± 2543	4078 ± 2936*	<0.001

*p-value<0.001, Paired t-test, *FT3* Free triiodothyronine, *FT4* Free thyroxine, *anti-TPO* anti-thyroid peroxidase antibody, *anti-TG* anti-thyroglobulin, *TSH* thyroid stimulating hormone, *TG* thyroglobulin, *hs-CRP* high sensitivity C-reactive protein

Thyroid medication consumption was reported by 15.8% of participants at program entry and another 3.3% of participants started taking thyroid medication between entry and follow-up. Using thyroid biomarkers, hypothyroidism was found in 1.8% of participants at baseline, which is similar to Canadian population estimates of 2% [32]. At follow-up 0.4% were classified as hypothyroid. After excluding participants who took thyroid medications at program entry or follow-up, or thyroid medications were initiated some times between entry and follow-up, 1.3% of participants were hypothyroid at program entry which decreased to 0.3% at follow-up. Subclinical hypothyroidism (SCH) was detected in 22.1% of participants at baseline and 5.8% at follow-up. Again, after excluding participants on thyroid medications at any point during the program, the incidence of SCH decreased from 21.7% at baseline to 6.1% at follow-up.

Among those participants who were hypothyroid at baseline, 33.3% were on thyroid medications at program entry and another 10% started taking thyroid medications at some point during follow-up. Thyroid medication consumption was reported by 21.3% of subclinical hypothyroid participants with an additional 6.6% starting medication later in the program. However, data for medication doses and any change in doses over time is not available in the current study, due to poor patient recall.

Of those with SCH, 91% had anti-TG antibody titers and 36% had anti-TPO antibody titers above the reference range. In addition, 26% of SCH had inflammation (hs-CRP ≥ 3 mg/L). Participants who presented thyroid symptoms like weight gain, cool body temperature, low mood, brain fog and refreshing sleep, had significantly higher T4 levels compared to those who did not present the symptoms. Among those participants who had high TSH levels at baseline (≥3 mlU/L), there was a significant negative correlation between T4 and the majority of hypothyroid symptoms, revealing that with increasing T4 levels, the incidence of presenting brain fog (*r* = −0.125), low mood (*r* = −0.120), unrefreshing sleep (*r* = −0.133), cool body temperature (*r* = −0.060) and weight gain (*r* = −0.102) were significantly decreased.

Participants were considered at-risk for autoimmune thyroid disease (ATD) when anti-thyroid antibody levels were above the reference range. Considering anti-TPO, 32% were at-risk at baseline which decreased to 20% at follow-up. For anti-TG, 93% of participants were at-risk at baseline down to 21% at follow-up. Concomitant high levels of anti-TPO and anti-TG were present in 29% of participants at baseline and 9% at follow-up. Overall, for those at-risk for ATD at baseline, more than 60% were no longer considered at-risk at follow-up. In contrast, for those who had normal levels of antibodies at baseline, the chance of being at risk for ATD at follow-up was 1%.

After 1 year in program, mean serum 25(OH)D concentrations significantly increased, from 78 ± 34 nmol/L to 110 ± 22 nmol/L, and was consistent with the increase in vitamin D supplementation dose, from 1436 ± 2543 IU/d to 4078 ± 2936 IU/d at follow-up (Table 2). At follow-up serum 25(OH)D levels ≥ 100 nmol/L were achieved by 86% of participants with a mean intake of 3940 ± 2660 IU/d. Moreover, 11% had serum 25(OH)D levels ≥ 125 nmol/L with a mean intake of 6164 ± 4398 IU/d.

Subclinical hypothyroid cases (SCH) were investigated in comparison with hypothyroid patients and participants with normal thyroid function. Following significant increase in serum 25(OH)D levels, we found significant improvements in thyroid antibodies and TSH, with no change in thyroid hormones for SCH (Supplementary Table 2). This improvement was more pronounced in hypothyroid patients rather than SCH cases.

#### Vitamin D, thyroid measures, and inflammation

We examined the risk of autoimmune thyroid disease and hypothyroidism with respect to measurable changes in 25(OH)D levels to investigate whether thyroid measure improvements were associated with the intervention program. The relative risks for increased levels of anti-TPO, anti-TG, and inflammation (hs-CRP) were found to be significantly lower when serum 25(OH)D levels ≥ 125 nmol/L were achieved. Serum 25(OH)D concentrations <125 nmol/L were associated with an increased risk of thyroid disease, a 115% increased risk of elevated anti-TG antibody, 118% increased risk of anti-TPO antibody and 107% increased risk of elevated TSH. Serum 25(OH)D levels above 125 nmol/L were associated with 60 and 14% less chance of having low levels of thyroid hormones (FT4 and FT3) (Table 3).

**Table 3:** Relative risk (RR) for thyroid condition worsening according to serum 25(OH)D level and vitamin D supplementation dose at one-year follow-up

	Relative risk (95% CI) based on serum 25(OH)D, nmol/L [*n* = 11,017]			Relative risk (95% CI) based on vitamin D supplement dose, IU/d [*n* = 11,017]		
	<125 (*n* = 9796)	≥125 (*n* = 1226)	*P* value^a^	<4000 (*n* = 8215)	≥4000 (*n* = 2806)	*P* value^a^
FT3 decrease	1.02 (1.002–1.029)	0.88 (0.793–0.983)	0.02	1.02 (1.003–1.049)	0.93 (0.869–0.991)	0.03
FT4 decrease	1.56 (1.377–1.773)	0.95 (0.938–0.963)	0.01	1.39 (1.292–1.501)	0.90 (0.882–0.921)	<0.001
Anti-TPO increase	1.18 (1.158–1.202)	0.32 (0.291–0.360)	<0.001	1.60 (1.545–1.653)	0.34 (0.316–0.358)	<0.001
Anti-TG increase	1.15 (1.132–1.172)	0.37 (0.331–0.409)	<0.001	1.49 (1.445–1.536)	0.37 (0.352–0.399)	<0.001
TSH increase	1.07 (1.058–1.087)	0.55 (0.495–0.621)	<0.001	1.20 (1.172–1.224)	0.57 (0.536–0.615)	<0.001
TG increase	1.02 (0.998–1.035)	0.89 (0.776–1.015)	0.08	1.04 (1.007–1.071)	0.90 (0.832–0.978)	0.01
hs-CRP increase	1.12 (1.099–1.132)	0.43 (0.385–0.479)	<0.001	1.28 (1.245–1.309)	0.51 (0.477–0.543)	<0.001

*FT3* Free triiodothyronine, *FT4* Free thyroxine, *anti-TPO* anti-thyroid peroxidase antibody, *anti-TG* anti-thyroglobulin, *TSH* thyroid stimulating hormone, *TG* thyroglobulin, *hs-CRP* high sensitivity C-reactive protein

^a^ Chi Square test

**Fig. 1:**
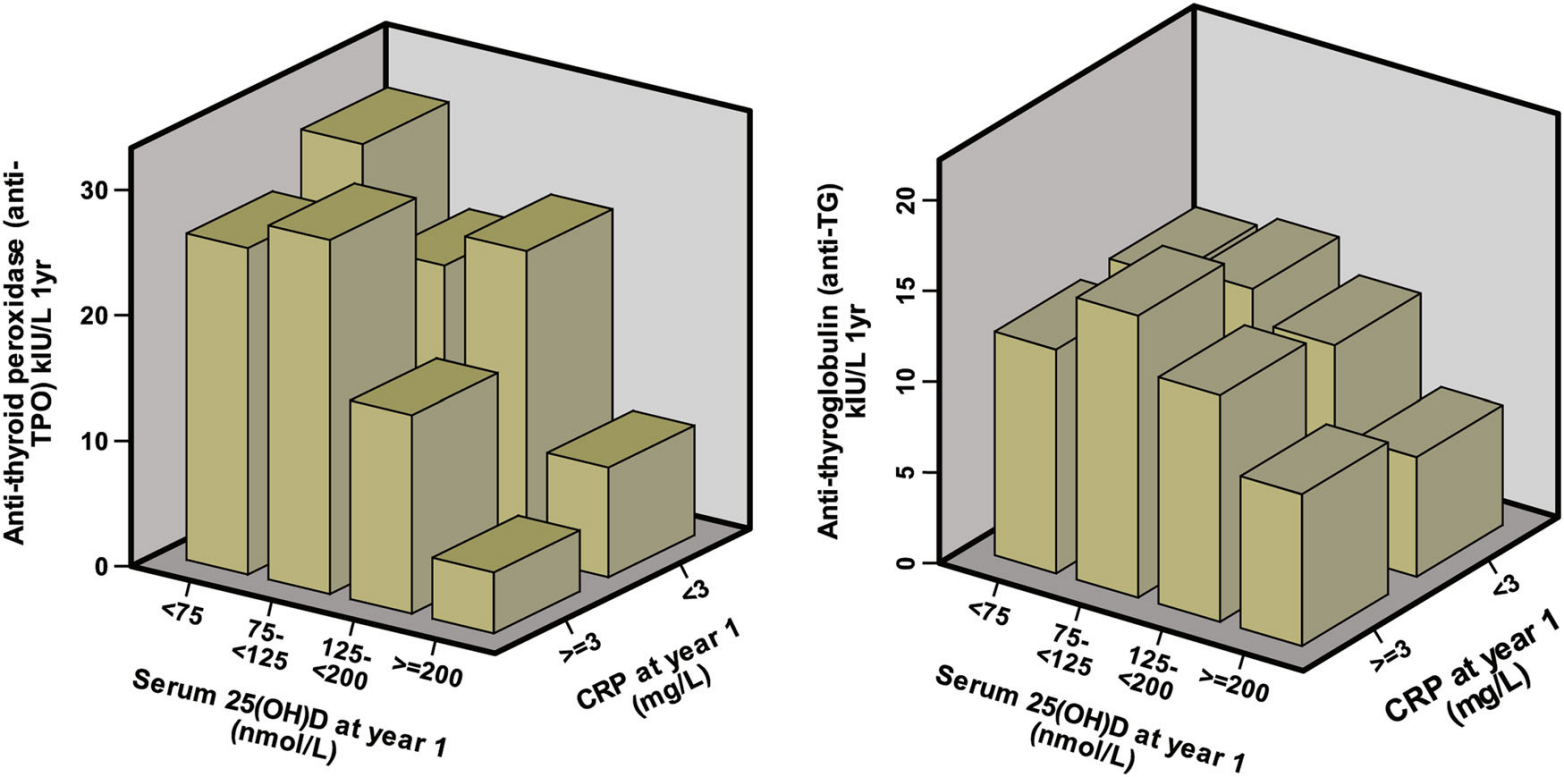
Relationship between anti-thyroid antibody levels, serum 25(OH)D and C reactive protein (hs-CRP). Left Panel, anti-TPO; Right Panel, anti-TG

Inflammation (hs-CRP >3 mg/L) was present in 17% of participants with high anti-TG, 8% with high anti-TPO and 6% of participants with both anti-TG and anti-TPO above the reference range (Fig. 1).

Thyroid assessment questionnaire was completed by *n* = 3367 participants at entry to the program and again 1 year follow-up. Both vitamin D supplementation dose and serum 25(OH)D levels were found to significantly reduce the risk of reported hypothyroid symptoms at follow-up (Table 4). The relative risk of reporting brain fog, low mood, unrefreshing sleep, weight gain or low energy was significantly higher in participants whose serum 25(OH)D level were <125 nmol/L after 1 year in program, compared to those with serum 25(OH)D levels ≥125 nmol/L. Vitamin D supplementation dose ≥4000 IU/d was associated with lower risk of reporting brain fog, low mood, unrefreshing sleep, weight gain, and low energy.

**Table 4:** Relative risk for reported thyroid symptoms in accordance to serum 25(OH)D level and vitamin D supplementation dose at one-year follow-up

	Relative risk based on serum 25(OH)D, nmol/L (*n* = 3367)			Relative risk based on vitamin D supplement dose, IU/d (*n* = 3367)		
	<125 (*n* = 2389)	≥125 (*n* = 978)	*P* value^a^	<4000 (*n* = 1001)	≥4000 (*n* = 2366)	*P* value^a^
Brain fog	1.053 (1.007–1.10)	0.88 (0.783–0.987)	0.03	1.13 (1.012–1.25)	0.95 (0.905–0.996)	0.03
Macroglossia	1.109 (1.023–1.204)	0.75 (0.571–0.977)	0.02	1.09 (0.866–1.34)	0.97 (0.877–1.07)	0.5
Low mood	1.09 (1.042–1.144)	0.79 (0.698–0.903)	<0.001	1.2 (1.07–1.34)	0.92 (0.875–0.972)	0.002
Unrefreshing sleep	1.09 (1.046–1.14)	0.80 (0.719–0.895)	<0.001	1.18 (1.06–1.305)	0.93 (0.89–0.97)	0.002
Cool body temperature	0.99 (0.947–1.045)	1.012 (0.903–1.135)	0.8	1.00 (0.895–1.13)	0.99 (0.95–1.05)	0.9
Weight gain	1.18 (1.12–1.23)	0.62 (0.522–0.733)	<0.001	1.27 (1.13–1.44)	0.89 (0.835–0.95)	<0.001
Low energy	1.35 (1.18–1.548)	0.89 (0.855–0.935)	<0.001	1.05 (1.01–1.106)	0.89 (0.794–0.999)	0.05

^a^ Chi square test

### Nested case–control study

#### Baseline characteristics

To examine the relationship between serum 25(OH)D status and improved thyroid function we compared, in a 1:4 ratio, hypothyroid cases (*n* = 103) to euthyroid controls (*n* = 412) matched based on age, sex, BMI and the first two digits of their postal code. Hypothyroid cases were defined based on their measured thyroid biomarkers (TSH, FT3, and FT4). Participants taking thyroid medication at program entry or follow-up (desiccated thyroid and synthroid) were excluded. Intervention between baseline and follow-up included vitamin D and multivitamin package. As expected hypothyroid individuals had higher levels of TSH, anti-TPO, and anti-TG, lower levels of FT3 and FT4, and more frequently reported brain fog, unrefreshing sleep, and weight gain (Supplementary Table 3). The reported history of vitamin and supplement use was not significantly different between hypothyroid and control groups. However, at baseline, serum 25(OH)D was significantly lower in cases than controls (68 ± 32 vs. 82 ± 34 nmol/L; *p*-value <0.001).

#### Biomarker changes

Serum 25(OH)D concentrations increased to a greater extent in hypothyroid cases compared to the controls (mean change 42 ± 26 vs. 28 ± 31 nmol/L, respectively, *p*-value <0.001), whereas vitamin D supplementation doses were lower at 3517 ± 2620 IU/d in cases compared to 4150 ± 3321 IU/d in controls (*p*-value 0.05). Among hypothyroid cases optimal 25(OH)D concentrations (>100 nmol/L) were achieved in 92% at follow-up, up from 15% at baseline, whereas 80% of controls achieved optimal levels (up from 26%). In comparison with controls, cases had significantly greater decreases in levels of TSH, anti-TPO, anti-TG, and greater increase in thyroid hormones concentrations (FT4 and FT3) (Table 5). Hypothyroid cases (*n* = 69) that were vitamin D insufficient at baseline had greater decrease in anti-TPO (−144 ± 164 vs. −2.7 ± 71), TSH (−4.0 ± 4.8 vs. 0.2 ± 0.7) and greater increase in FT4 (4.5 ± 1.4 vs. −1.2 ± 2.9) in comparison with those who were vitamin D sufficient (*n* = 34) [anti-TPO (−68 ± 103 vs. −3 ± 71), TSH (−2.3 ± 2.9 vs. 0.2 ± 0.8) and FT4 (3.4 ± 1.4 vs. −0.3 ± 0.7)].

**Table 5:** Comparison of thyroid measures over time between cases and controls

Serum parameter	Case			Control			Between groups comparison (*P*-value)^a^
	*N*	Baseline (mean ± SD	Follow-up (mean ± SD)	*N*	Baseline (mean ± SD)	Follow-up (mean ± SD)	
FT3 (pmol/L)	103	4.34 ± 0.6	4.57 ± 0.2^b^	412	4.85 ± 0.7	4.30 ± 0.5^b^	<0.001
FT4 (pmol/L)	103	9.06 ± 1.1	13.4 ± 1.0^b^	412	14.7 ± 2.8	13.5 ± 1.0^b^	<0.001
Anti-TPO (kU/L)	103	162.8 ± 120	43.4 ± 116^b^	412	21.5 ± 54.8	18.8 ± 26.7	<0.001
Anti-TG (kU/L)	103	51.8 ± 33.7	17.6 ± 27.2^b^	412	32.0 ± 61.3	14.5 ± 30.7^b^	0.05
TSH (mlU/L)	103	5.97 ± 4.2	2.50 ± 1.1^b^	412	1.78 ± 0.7	1.98 ± 0.6^b^	<0.001
TG (µg/L)	31	43.1 ± 39.4	25.6 ± 4.0^b^	244	28.2 ± 26.3	25.4 ± 5.9	0.003
hs-CRP (mg/L)	103	2.05 ± 2.2	2.52 ± 1.4	412	2.35 ± 3.1	2.41 ± 1.9	0.2
25(OH)D (nmol/L)	103	68 ± 32	110 ± 14^b^	412	82 ± 34	110 ± 27^b^	0.001
Vitamin D dose (IU/day)	103	1243 ± 2606	3517 ± 2620^b^	412	1824 ± 2770	4150 ± 3321^b^	0.9

^a^ Independent samples *T*-test (between groups comparison)

^b^ *P *< 0.05 paired samples *T*-test (within group comparison)

We also compared hypothyroid cases who were vitamin D deficient [serum 25(OH)*D* < 75 nmol/L] and not taking thyroid medication with a hypothyroid control group who were vitamin D sufficient [serum 25(OH)*D* ≥ 75 nmol/L] and on no medication, in a ratio of 1:4, age-matched, sex-matched, and BMI-matched (Supplementary Table 4). At program entry, serum FT4 was significantly lower and anti-TPO and anti-TG levels were significantly higher in hypothyroid cases who were vitamin D deficient compared to control group. After 1 year follow-up, hypothyroid cases who were vitamin D deficient had less of a decrease in FT4 (−0.12 ± 2.7 vs. −1.1 ± 2.3), and a greater decrease in anti-TPO (−28.1 ± 83.9 vs. −13.1 ± 77.8) and anti-TG (−84.5 ± 107 vs. −37.9 ± 118.7). Further, cases had a significantly greater increase in serum 25(OH)D level (47.8 ± 20.8 vs. 12.0 ± 33.7) and greater decrease in hs-CRP (−0.97 ± 7.0 vs. −0.06 ± 4.0) than controls.

#### Relationship between vitamin D status and thyroid function

After 1 year in program, changes in thyroid measures differed significantly between hypothyroid cases compared to controls (Table 5). Larger decreases in TSH, anti-TPO, and anti-TG were found for cases. Among vitamin D-deficient cases, an increased serum 25(OH)*D* ≥ 50 nmol/L was associated with greater reductions in biochemical signs of hypothyroidism and thyroid autoimmune disease including an increase in FT4 and FT3, and large decrease in serum TSH, anti-TPO, anti-TG, and TG (Supplementary Table 5).

We utilized binary logistic regression to determine the effect of serum 25(OH)D on changes in thyroid measures (Supplementary Table 6). Thyroid measures were corrected for age, sex, BMI, season of observation and thyroid medication use. Regression analysis revealed that serum 25(OH)D improvement to above 75 nmol/L had a significant positive association with decreased serum TSH (*β* = 1.135, 95% CI 1.002–1.353), decreased anti-TPO (*β* = 1.950, 95% CI 1.351–2.815), decreased anti-TG (*β* = 1.445, 95% CI 1.002–2.091) and increased FT4 (*β* = 1.413, 95% CI 1.006–2.129) levels. Moreover, serum vitamin B12 improvement had a significant association with increased serum FT4 (*β* = 1.737, 95% CI 1.387–2.176) and increased FT3 (*β* = 1.469, 95% CI 1.203–1.794) levels. Serum TSH level varied seasonably with significantly lower levels during the winter season. These changes were independent of changes affecting FT3 and FT4.

## Discussion

Approximately 2% of participants in this health and wellness program were found to be hypothyroid at program entry, with an additional 22% classified as subclinical hypothyroid. High incidence of subclinical hypothyroidism in this study population might explain 15.8% of participants that reported thyroid medication use. Like other studies [25, 42, 43], we found that hypothyroid individuals were three times more likely (27%) and subclinical hypothyroidism nearly twice as likely (17%) to be vitamin D-deficient than euthyroid individuals (10%). Supplementation with vitamin D resulted in an overall reduction in TSH and in the detection of hypothyroidism (down 58% at follow-up). Most intriguing was the finding that subclinical hypothyroidism was reduced by 72% at follow-up. It is well accepted that subclinical hypothyroidism is a mild, early form of thyroid failure [44]. Achieving serum 25(OH)D concentrations above 125 nmol/L reduced the risk for high TSH as well as symptoms of low thyroid function (brain fog, weight gain, low mood, unrefreshing sleep and low energy). These results are consistent with clinical trials centered on patients with autoimmune thyroid diseases showing that thyroid antibodies decreased significantly following vitamin D supplementation compared to patients receiving no vitamin D [27, 29]. In combination with these studies, our findings suggest that vitamin D may influence thyroid function and that supplementation may be used as an intervention to help prevent hypothyroidism. We also found that 76% of hypothyroid patients were vitamin B12 insufficient (serum vitamin B12 <450 pmol/L) and improving serum vitamin B12 status was significantly associated with increased thyroid hormones (FT3 and FT4). Replacement of B12 might lessen hypothyroid symptoms. Jabbar et al. [45] and Al-Khamis [46] previously showed that there is a high prevalence of vitamin B12 deficiency in hypothyroid patients and replacing vitamin B12 improves their symptoms.

The current study revealed that serum TSH is significantly affected by season and is the highest in winter, when average serum 25(OH)D concentrations were at their lowest. Moreover, this association was independent of thyroid hormones and yet was dependent on improvements in serum 25(OH)D status. Considering the high prevalence of vitamin D deficiency worldwide and the high incidence of undiagnosed subclinical hypothyroidism in the general population (as we found in Canadians), the existence of the association between TSH and vitamin D status is of high importance and makes vitamin D supplementation a potential asset for patients already taking thyroid medications.

Autoimmune thyroid disease (AITD), including Grave’s disease and Hashimoto’s thyroiditis, are prevalent autoimmune disorders affecting an estimated 5% of the population [47]. A link between hypovitaminosis D and thyroid autoimmunity has been established [23] and a review of 20 case–control studies revealed that lower levels of 25(OH)D were prevalent in autoimmune thyroid diseases [18]. We found elevated anti-thyroid antibodies, both anti-TPO and anti-TG, in 29% of the population considered at-risk for developing autoimmune thyroid disease, over 80% of whom did not have optimal 25(OH)D levels (>100 nmol/L) at baseline.

Proper thyroid function requires appropriate physiological levels of serum 25(OH)D (i.e., 100–130 nmol/L) [16]. It has been suggested that physiological levels should be sustained for a considerable period of time (e.g., 2–3 years) for the goal of chronic disease prevention or treatment achieved [48]. In accordance, we also found that of the subjects who achieved serum 25(OH)D above 100 nmol/L at follow-up, roughly 1 year after program entry, only 8.8% were still considered at-risk of AITD. Given that AITD is the main cause of thyroid dysfunction in Canada [49], the remarkable decrease in thyroid autoantibodies following improved serum 25(OH)D status might explain the significant decrease in the prevalence of hypothyroidism (from 2 to 0.4%) and this is likely to attributable to the immunoregulatory role of vitamin D rather than a direct effect of vitamin D on thyroid function. Short duration of supplementation and low serum 25(OH)D levels (rather than the physiological levels) are likely reasons why the effects of vitamin D on thyroid function were not recovered in other studies [27]. Improved serum 25(OH)D status also significantly affected inflammation by decreasing hs-CRP which may provide a potential reason why improving 25(OH)D status promotes thyroid function. Given vitamin D’s extensive roles in immune cell function and inflammation, these results are not surprising. Supplementation with vitamin D has been found to induce tolerance [50, 51] and reduce auto reactivity in other autoimmune conditions such as multiple sclerosis [15, 52].

We utilized a nested case–control study design to further investigate the associations between thyroid function and vitamin D. Hypothyroid cases not taking thyroid medication had reduced TSH levels by 58% with a mean level that was within the reference range at follow-up. Large reductions in anti-thyroid antibody levels were found in cases with decreases in anti-TG by 66% and anti-TPO by 73%. Changes in thyroid hormones and TSH were significantly correlated with improvement in hypothyroid symptoms assessed through thyroid assessment questionnaire and are clinically significant. Vitamin D deficient cases experienced greater reductions in biochemical signs of hypothyroidism and autoimmune thyroiditis. Vitamin D deficiency appeared to be a relevant risk factor for hypothyroidism and autoimmune thyroid disease, in addition to which supplementation with vitamin D provided measurable benefit.

The limitations of the study include the retrospective nature of the analyzes. Because the sample was drawn from a community-based program there is a selection bias to contend with, yet the extremely large sample size (over 11,000) must be considered a strength. Some risk factors associated with thyroid disease, such as cigarette smoking, were not available for all participants. The main strength of this study lies in the large number of thyroid function tests that were analyzed longitudinally to investigate the relationship between serum 25(OH)D status and these parameters.

## Conclusion

Overall, the results of the current study suggest that for normal thyroid function an optimal 25(OH)D concentration above 100–125 nmol/L may be required. Although improving other nutrient status, like vitamin B12, should also be taken into consideration. Of concern, recommended daily intakes for vitamin D are aimed at achieving serum 25(OH)D concentrations of 50 nmol/L and targeted at bone health alone. Vitamin D offers a safe and economical approach to improve thyroid function and may provide protection from developing thyroid disease.

## Electronic supplementary material

Supplementary material
