# Supplementary material for: Physiological serum 25-hydroxyvitamin D concentrations are associated with improved thyroid function—observations from a community-based program
Source: Endocrine. 2017 Oct 24;58(3):563–73. doi: 10.1007/s12020-017-1450-y (PMC5693977; doi:10.1007/s12020-017-1450-y)
Supplement: Supplementary file 1 — Supplementary material [file 12020_2017_1450_MOESM1_ESM.docx]

**Supplementary materials**

**Table S1.** Comparison of demographics between baseline and follow-up

| **Parameter** | **Baseline N (%)** | **Follow-up N (%)** | **P value** |
| --- | --- | --- | --- |
| **Age, years** | 11,017 48 ± 16 (18-95 years) | --------- | -------- |
| **Gender**  Female  Male | 11,017  6,378 (58)  4,649 (42) | --------- | -------- |
| **Body Mass Index, kg/m^2^**  Normal weight (18.5-24.99)  Overweight (25-29.99)  Obese (≥ 30) | 11,017  3,999 (36.3)  4,055 (36.8)  2,964 (26.9) | 11,017  3,900 (35.4)  4,110 (37.3)  3,008 (27.3) | 0.7 |
| **Medication history**  Desiccated thyroid (Armor thyroid)  Synthroid  Other thyroid medications | 1,099  30 (3)  191 (17.4)  40 (3.6) | 1,099  47 (4.6)  209 (19)  40 (3.6) | 0.3 |
| **Supplementation history**  Iodine  Magnesium  Niacin  Vitamin D | 977 (19.3)/4,883  228 (9.7)/2,356  29 (1.7)/1,799  4,695 (42.6)/11,017 | 2,671 (54.3)/4,883  1366 (58%)/2,356  114 (6.5)/1,799  9,334 (84.4)/11,017 | 0.03  0.01  0.1  0.001 |
| **Thyroid Questionnaire**  Brain fog  Low energy level  Macroglossia  Low mood  Unrefreshing sleep  Cool body temperature  Weight gain | 1,182 (35.6)/3,316  1,076 (53.9)/1,996  389 (13)/2,995  1,002 (31.1)/3,221  1,566 (46.3)/3,385  1,033 (32.1)/3,215  794 (25.3)/3,144 | 1,084 (32.7)/3,316  727 (36.6)/1,996  183 (6.1)/2,995  880 (27.3)/3,221  1,379 (40.7)/3,385  943 (29.3)/3,215  583 (18.5)/3,144 | 0.1  0.04  0.03  0.06  0.04  0.1  0.04 |
| **Serum 25(OH)D status, nmol/L**  < 50  50 -100  100 – 150  150 -200  200 – 250  ≥ 250 | 11,017  2,102 (19.1)  6,661 (60.4)  1,867 (16.9)  288 (2.6)  76 (0.7)  23 (0.2) | 11,017  64 (0.6)  1,497 (13.6)  8,905 (80.8)  467 (4.2)  66 (0.6)  18 (0.2) | < 0.001 |

Age and BMI presented as Mean ± SD

**Table S2** Comparison of changes in thyroid measures over time between hypothyroid, subclinical hypothyroid patients and euthyroid participants

| **Parameter change** | | **N** | **Mean** | **SD** | **P value** |
| --- | --- | --- | --- | --- | --- |
|  |  |  |  |  |  |
| **FT3** | Euthyroidism | 8396 | -0.36^c^ | 0.83 | < 0.001 |
|  | Subclinical hypothyroidism | 2433 | -0.23^b^ | 0.67 |  |
|  | Hypothyroidism | 193 | 0.35^a^ | 0.72 |  |
| **FT4** | Euthyroidism | 8396 | -1.44^b^ | 3.03 | < 0.001 |
|  | Subclinical hypothyroidism | 2433 | -1.11^b^ | 2.22 |  |
|  | Hypothyroidism | 193 | 4.58^a^ | 1.59 |  |
| **Anti-TPO** | Euthyroidism | 8396 | -2.07^a^ | 65.5 | < 0.001 |
|  | Subclinical hypothyroidism | 2433 | -28.4^b^ | 117 |  |
|  | Hypothyroidism | 193 | -114^c^ | 106 |  |
| **Anti-TG** | Euthyroidism | 8396 | -16.7^a^ | 94 | < 0.001 |
|  | Subclinical hypothyroidism | 2433 | -54.2^c^ | 147 |  |
|  | Hypothyroidism | 193 | -38.2^b^ | 144 |  |
| **TSH** | Euthyroidism | 8396 | 0.20^a^ | 0.89 | < 0.001 |
|  | Subclinical hypothyroidism | 2433 | -2.13^b^ | 2.43 |  |
|  | Hypothyroidism | 193 | -4.72^c^ | 7.02 |  |
| **TG** | Euthyroidism | 4909 | -2.99^a^ | 24.4 | < 0.001 |
|  | Subclinical hypothyroidism | 1549 | -6.89^a^ | 30.3 |  |
|  | Hypothyroidism | 51 | -20.5^b^ | 47.3 |  |
| **CRP** | Euthyroidism | 8353 | -0.28 | 4.72 | 0.3 |
|  | Subclinical hypothyroidism | 2422 | -0.43 | 4.65 |  |
|  | Hypothyroidism | 193 | -0.33 | 4.50 |  |
| **Homocysteine** | Euthyroidism | 8340 | -1.17 | 3.04 | 0.08 |
|  | Subclinical hypothyroidism | 2413 | -1.31 | 3.04 |  |
|  | Hypothyroidism | 189 | -1.43 | 3.27 |  |
| **Serum 25(OH)D** | Euthyroidism | 8396 | 32.9^b^ | 35.8 | 0.004 |
|  | Subclinical hypothyroidism | 2433 | 31.2^b^ | 35.4 |  |
|  | Hypothyroidism | 193 | 40.0^a^ | 33.6 |  |
| **Vitamin D dose** | Euthyroidism | 8391 | 2645 | 3695 | 0.7 |
|  | Subclinical hypothyroidism | 2432 | 2643 | 3862 |  |
|  | Hypothyroidism | 193 | 2429 | 3730 |  |
| **Vitamin B12** | Euthyroidism | 8368 | 806 | 772 | 0.1 |
|  | Subclinical hypothyroidism | 2429 | 839 | 814 |  |
|  | Hypothyroidism | 193 | 776 | 567 |  |

FT3= Free triiodothyronine, FT4= Free thyroxine, anti-TPO= anti-thyroid, peroxidase antibody, anti-TG=anti-thyroglobulin, TSH=thyroid stimulating hormone, TG=thyroglobulin, hs-CRP=high sensitivity C-reactive protein

**Table S3.** Baseline characteristics of the hypothyroid patients (case) and matched control group (1:4 ratio) [participants who received thyroid medication were excluded from case-control analysis]

| **Parameter** | **Case (n=103)** | | | **Control (n=412)** | | | **P value** |
| --- | --- | --- | --- | --- | --- | --- | --- |
| **Gender**  Female  Male | 59 (57%)  44 (43%) |  |  | 235 (57%)  177 (43%) |  |  | 1^#^ |
| **Vitamin & Supplements**  Iodine (100 µg/d)  Magnesium  Niacin | 9 (8.8%)  2 (2.5%)  0 (0%) |  |  | 74 (18%)  20 (5.8%)  4 (1.2%) |  |  | 0.8^#^  0.3^#^  0.6^#^ |
| **Thyroid Assessment Questionnaire (TAQ)**  Brain fog  Macroglossia  Low mood  Unrefreshing sleep  Cool body temperature  Weight gain  Low Energy level | 45 (56.3%)  9 (13.0%)  38 (46.3%)  56 (64.4%)  28 (37.8%)  31 (41.3%)  22 (31.0%) |  |  | 151 (39.6%)  41 (11.4%)  145 (38.2%)  191 (49.4%)  113 (30.5%)  97 (26.3%)  86 (32.3%) |  |  | 0.006^#^  0.6^#^  0.1^#^  0.01^#^  0.2^#^  0.009^#^  0.8^#^ |
|  | **N** | **Mean** | **SD** | **N** | **Mean** | **SD** | **P value** |
| **Age (year)** | 103 | 49.5 | 13.2 | 412 | 49.6 | 13.9 | 0.9 |
| **BMI (kg/m2)** | 103 | 28.8 | 4.6 | 412 | 27.5 | 5.0 | 0.3^*^ |
| **Vitamin B12 pmol/L** | 103 | 363 | 319 | 412 | 445 | 264 | 0.007^*^ |
| **Serum 25(OH)D (nmol/L)** | 103 | 68 | 32 | 412 | 82 | 34 | <0.001^*^ |
| **Vitamin D (IU/d)** | 103 | 1,243 | 2,606 | 412 | 1,824 | 2,770 | 0.05^*^ |
| **hs-CRP (mg/L)** | 103 | 2.05 | 2.2 | 412 | 2.34 | 3.1 | 0.3^*^ |
| **Homocysteine (µmol/L)** | 103 | 10.1 | 3.5 | 412 | 9.98 | 3.1 | 0.6^*^ |
| **Serum FT3 (pmol/L)** | 103 | 4.34 | 0.58 | 412 | 4.85 | 0.71 | < 0.001^*^ |
| **Serum FT4 (pmol/L)** | 103 | 9.06 | 1.09 | 412 | 14.66 | 2.8 | < 0.001^*^ |
| **Anti-TPO (kU/L)** | 103 | 162.8 | 120 | 412 | 21.5 | 54.8 | < 0.001^*^ |
| **Anti-TG (kU/L)** | 103 | 51.8 | 33.7 | 412 | 32.0 | 61.3 | 0.07^*^ |
| **Serum TSH (mlU/L)** | 103 | 5.97 | 4.2 | 412 | 1.78 | 0.7 | < 0.001^*^ |
| **Serum TG (µg/L)** | 31 | 43.1 | 39.4 | 244 | 28.2 | 26.3 | 0.006^*^ |

#Chi square test, *Independent Samples T-test, FT3= Free triiodothyronine, FT4= Free thyroxine, anti-TPO= anti-thyroid peroxidase antibody, anti-TG=anti-thyroglobulin, TSH=thyroid stimulating hormone, TG=thyroglobulin, hs-CRP=high sensitivity C-reactive protein

**Table S4.** Comparison of thyroid measures over time between cases and controls

|  | **Hypothyroid & low vitamin D (Case)** | | **Hypothyroid & Normal vitamin D (Control)** | | **Between group comparison** |
| --- | --- | --- | --- | --- | --- |
|  | **N** | **Mean ± SD** | **N** | **Mean ± SD** | **p value^*^** |
| **FT3 (pmol/L)** |  |  |  |  |  |
| *Baseline* | 118 | 4.75 ± 0.63 | 472 | 4.77 ± 0.64 | 0.7 |
| *Follow-up* | 118 | 4.57 ± 0.22^#^ | 472 | 4.57 ± 0.26^#^ | 0.8 |
| *Change* | 118 | -0.18 ± 0.64 | 472 | -0.20 ± 0.64 | 0.7 |
| **FT4 (pmol/L)** |  |  |  |  |  |
| *Baseline* | 118 | 13.42 ± 2.5 | 472 | 14.52 ± 2.2 | < 0.001 |
| *Follow-up* | 118 | 13.30 ± 1.1 | 472 | 13.40 ± 1.0^#^ | 0.3 |
| *Change* | 118 | -0.12 ± 2.7 | 472 | -1.1 ± 2.3 | < 0.001 |
| **Anti-TPO (kU/L)** |  |  |  |  |  |
| *Baseline* | 118 | 60.8 ± 127.0 | 472 | 38.1 ± 96.4 | 0.03 |
| *Follow-up* | 118 | 32.7 ± 82.1^#^ | 472 | 24.9 ± 64.8^#^ | 0.2 |
| *Change* | 118 | -28.1 ± 83.9 | 472 | -13.1 ± 77.8 | 0.05 |
| **Anti-TG (kU/L)** |  |  |  |  |  |
| *Baseline* | 118 | 103.2 ± 117.7 | 472 | 51.3 ± 117.9 | <0.001 |
| *Follow-up* | 118 | 18.7 ± 43.8^#^ | 472 | 13.3 ± 31.3^#^ | 0.1 |
| *Change* | 118 | -84.5 ± 107.0 | 472 | -37.9 ± 118.7 | 0.001 |
| **TSH (mlU/L)** |  |  |  |  |  |
| *Baseline* | 118 | 4.34 ± 1.2 | 472 | 4.32 ± 1.5 | 0.9 |
| *Follow-up* | 118 | 2.68 ± 1.1^#^ | 472 | 2.64 ± 1.0^#^ | 0.6 |
| *Change* | 118 | -1.66 ± 1.4 | 472 | -1.68 ± 1.6 | 0.8 |
| **TG (µg/L)** |  |  |  |  |  |
| *Baseline* | 74 | 30.6 ± 30.2 | 314 | 33.0 ± 34.4 | 0.5 |
| *Follow-up* | 74 | 26.9 ± 18.3 | 314 | 25.3 ± 5.7^#^ | 0.1 |
| *Change* | 74 | -3.7 ± 30.3 | 314 | -7.7 ± 33.2 | 0.6 |
| **hs-CRP (mg/L)** |  |  |  |  |  |
| *Baseline* | 114 | 2.21 ± 2.3 | 466 | 2.07 ± 2.7 | 0.6 |
| *Follow-up* | 114 | 1.24 ± 1.4 | 466 | 2.01 ± 1.8 | 0.05 |
| *Change* | 114 | -0.97 ± 7.0 | 466 | -0.06 ± 4.0 | 0.05 |
| **25(OH)D (nmol/L)** |  |  |  |  |  |
| *Baseline* | 118 | 58.9 ± 13.0 | 472 | 105.1 ± 27.6 | < 0.001 |
| *Follow-up* | 118 | 106.7 ± 16.6^#^ | 472 | 117.1 ± 26.2^#^ | < 0.001 |
| *Change* | 118 | 47.8 ± 20.8 | 472 | 12.0 ± 33.7 | < 0.001 |
| **Vitamin D dose (IU/d)** |  |  |  |  |  |
| *Baseline* | 118 | 1104 ± 2364 | 472 | 2382 ± 2727 | < 0.001 |
| *Follow-up* | 118 | 4797 ± 2193^#^ | 472 | 5561 ± 2821^#^ | 0.006 |
| *Change* | 118 | 3693 ± 2990 | 472 | 3179 ± 3613 | 0.1 |

^#^ p<0.05 Paired Samples T-test (within group comparison); ^*^Independent Samples T-test (between groups comparison)

**Table S5** Association between thyroid measures and inflammatory marker changes with serum 25(OH)D level improvement at follow-up in hypothyroid cases (n=103) and control group (n=412) (1:4 ratio)

| **Changes** | **25(OH)D improve < 25 nmol/L (n=216)** | | | | **25(OH)D improve 25-50 nmol/L (n=141)** | | | | **25(OH)D improve ≥ 50 nmol/L (n=158)** | | | |
| --- | --- | --- | --- | --- | --- | --- | --- | --- | --- | --- | --- | --- |
|  | **Case** | | **Control** | | **Case** | | **Control** | | **Case** | | **Control** | |
|  | **N** | **Diff (SD)** | **N** | **Diff (SD)** | **N** | **Diff (SD)** | **N** | **Diff (SD)** | **N** | **Diff (SD)** | **N** | **Diff (SD)** |
| **25(OH)D (nmol/L)** | 25 | -5.5 (24.5) | 191 | -2.4 (25.8) | 29 | 38.3 (7.2) | 112 | 36.9 (7.6) | 49 | 68.3^*^ (12.9) | 109 | 71.3^#$^ (24.1) |
| **Vitamin D dose (IU/d)** | 25 | 1861 (2766) | 191 | 1799 (4624) | 29 | 1854 (3678) | 112 | 2534 (3448) | 49 | 2733 (3107) | 109 | 3033^#$^ (4325) |
| **FT3 (pmol/L)** | 25 | 0.21 (0.58) | 191 | -0.22 (0.67) | 29 | 0.33 (0.59) | 112 | -0.22 (0.72) | 49 | 0.38^*^ (0.60) | 109 | -0.40^ (0.71) |
| **FT4 (pmol/L)** | 25 | 3.4 (1.6) | 191 | -1.2 (2.8) | 29 | 4.5 (1.0) | 112 | -0.7 (2.9) | 49 | 4.7^*^  (1.4) | 109 | -1.6^#^^^$^ (2.9) |
| **Anti-TPO (kU/L)** | 25 | -44.3 (68.4) | 191 | -2.5 (74.8) | 29 | -106.1 (96.4) | 112 | -1.6 (61.8) | 49 | -165.6^*^ (149.6) | 109 | -4.1^#^^^$^ (75.3) |
| **Anti-TG (kU/L)** | 25 | -2.7 (59.4) | 191 | -21.0 (87.7) | 29 | -22.6 (122.2) | 112 | -22.0 (111.3) | 49 | -57.1^*^ (160.7) | 109 | -6.6^#^^ (60.2) |
| **TSH (mlU/L)** | 25 | -2.7 (3.1) | 191 | 0.11 (0.75) | 29 | -2.4 (2.5) | 112 | 0.20 (0.70) | 49 | -4.5^*^  (5.4) | 109 | 0.34^#^^^$^ (0.81) |
| **TG (µg/L)** | 10 | -18.8 (33.4) | 113 | -0.47 (23.8) | 6 | 6.3 (5.5) | 65 | -2.5 (27.1) | 15 | -26.1^*^ (45.5) | 66 | -7.3^#^^^$^ (20.3) |

FT3= Free triiodothyronine, FT4= Free thyroxine, anti-TPO= anti-thyroid, peroxidase antibody, anti-TG=anti-thyroglobulin, TSH=thyroid stimulating hormone, TG=thyroglobulin, hs-CRP=high sensitivity C-reactive protein

All superscripts indicates significant difference between cases and controls, Significant level p<0.05, *Within group (cases) comparison: all significant, except D dose, ^#^Within group (controls) comparison: all significant, except FT3, ^Between groups (case-control) comparison: all significant except serum D and D dose, ^$^Between groups (25(OH)D categories) comparison: all significant, except FT3, anti-TG

**Table S6.** Binary Logistic Regression demonstrating factors associated with thyroid measures improvement over one year follow-up

| **Thyroid measure improvement** |  | **B** | **Exp (B)** | **95% CI for Exp (B)** |
| --- | --- | --- | --- | --- |
| **FT4 (pmol/L)**  **R^2^ = 0.065**  **P < 0.001** | **Age*** | -0.013 | 0.987 | 0.979-0.994 |
|  | **Gender (Male)** | 0.029 | 1.030 | 0.819-1.294 |
|  | **25(OH)D improve (No improve)** | Ref |  |  |
|  | **<25 nmol/L** | 0.099 | 1.104 | 0.788-1.547 |
|  | **25-<50 nmol/L** | 0.288 | 1.334 | 0.961-1.852 |
|  | **50-<75 nmol/L*** | 0.422 | 1.525 | 1.061-2.192 |
|  | **≥ 75 nmol/L*** | 0.346 | 1.413 | 1.006-2.129 |
|  | **Vitamin B12 (BL≥450 & FU≥450 pmol/L)** | Ref |  |  |
|  | **Vitamin B12 (BL<450 & FU≥450 pmol/L)*** | 0.552 | 1.737 | 1.387-2.176 |
|  | **Thyroid medication (yes)** | -0.254 | 0.776 | 0.575-1.046 |
|  | **Season (Summer)** | Ref |  |  |
|  | **Season (Winter)** | 0.038 | 1.039 | 0.838-1.288 |
|  | **BMI kg/m^2^ (Normal weight)** | Ref |  |  |
|  | **Overweight*** | 0.603 | 1.828 | 1.368-2.444 |
|  | **Obese*** | 0.382 | 1.466 | 1.044-2.058 |
| **FT3 (pmol/L)**  **R^2^ = 0.079 P < 0.001** | **Age*** | 0.011 | 1.011 | 1.004-1.018 |
|  | **Gender (Male)*** | -0.645 | 0.525 | 0.423-0.650 |
|  | **25(OH)D improve (No improve)** | Ref |  |  |
|  | **<25 nmol/L** | -0.037 | 0.964 | 0.722-1.286 |
|  | **25-<50 nmol/L** | 0.048 | 1.049 | 0.787-1.399 |
|  | **50-<75 nmol/L** | 0.011 | 1.011 | 0.727-1.404 |
|  | **≥ 75 nmol/L** | -0.001 | 0.999 | 0.687-1.453 |
|  | **Vitamin B12 (BL≥450 & FU≥450 pmol/L)** | Ref |  |  |
|  | **Vitamin B12 (BL<450 & FU≥450 pmol/L)*** | 0.385 | 1.469 | 1.203-1.794 |
|  | **Thyroid medication (yes)*** | 0.713 | 2.041 | 1.587-2.625 |
|  | **Season (Summer)** | Ref |  |  |
|  | **Season (Winter)** | 0.036 | 1.037 | 0.854-1.259 |
|  | **BMI kg/m^2^ (Normal weight)** | Ref |  |  |
|  | **Overweight** | 0.043 | 1.044 | 0.819-1.329 |
|  | **Obese*** | -0.276 | 0.759 | 0.569-0.998 |
| **TSH (mlU/L)**  **R^2^ = 0.027**  **P < 0.001** | **Age*** | 0.012 | 1.012 | 1.005-1.019 |
|  | **Gender (Male)** | 0.154 | 1.167 | 0.945-1.441 |
|  | **25(OH)D improve (No improve)** | Ref |  |  |
|  | **<25 nmol/L** | -0.076 | 0.926 | 0.695-1.236 |
|  | **25-<50 nmol/L** | 0.010 | 1.011 | 0.758-1.348 |
|  | **50-<75 nmol/L** | -0.047 | 0.955 | 0.688-1.324 |
|  | **≥ 75 nmol/L*** | 0.126 | 1.135 | 1.002-1.353 |
|  | **Vitamin B12 (BL≥450 & FU≥450 pmol/L)** | Ref |  |  |
|  | **Vitamin B12 (BL<450 & FU≥450 pmol/L)** | -0.118 | 0.889 | 0.729-1.084 |
|  | **Thyroid medication (yes)** | -0.162 | 0.850 | 0.659-1.096 |
|  | **Season (Summer)** | Ref |  |  |
|  | **Season (Winter)*** | -0.201 | 0.818 | 0.674-0.993 |
|  | **BMI kg/m^2^ (Normal weight)** | Ref |  |  |
|  | **Overweight*** | -0.418 | 0.658 | 0.514-0.844 |
|  | **Obese** | -0.122 | 0.885 | 0.660-1.187 |
| **Anti-TPO (kU/L)**  **R^2^=0.073**  **P<0.001** | **Age*** | 0.010 | 1.010 | 1.003-1.016 |
|  | **Gender (Male)*** | -0.288 | 0.750 | 0.610-0.922 |
|  | **25(OH)D improve (No improve)** | Ref |  |  |
|  | **<25 nmol/L** | 0.214 | 1.239 | 0.932-1.646 |
|  | **25-<50 nmol/L** | 0.261 | 1.298 | 0.977-1.724 |
|  | **50-<75 nmol/L*** | 0.301 | 1.351 | 1.001-1.868 |
|  | **≥ 75 nmol/L*** | 0.668 | 1.950 | 1.351-2.815 |
|  | **Vitamin B12 (BL≥450 & FU≥450 pmol/L)** | Ref |  |  |
|  | **Vitamin B12 (BL<450 & FU≥450 pmol/L)** | -0.019 | 0.981 | 0.807-1.192 |
|  | **Thyroid medication (yes)** | 0.568 | 1.764 | 1.369-2.274 |
|  | **Season (Summer)** | Ref |  |  |
|  | **Season (Winter)** | -0.041 | 0.960 | 0.793-1.162 |
|  | **BMI kg/m^2^ (Normal weight)** | Ref |  |  |
|  | **Overweight*** | -0.634 | 0.530 | 0.418-0.673 |
|  | **Obese** | -0.023 | 0.977 | 0.739-1.291 |
| **Anti-TG (kU/L)**  **R^2^ = 0.100**  **P < 0.001** | **Age*** | 0.014 | 1.014 | 1.007-1.021 |
|  | **Gender (Male)*** | -0.312 | 0.732 | 0.595-0.900 |
|  | **25(OH)D improve (No improve)** | Ref |  |  |
|  | **<25 nmol/L** | 0.161 | 1.175 | 0.883-1.564 |
|  | **25-<50 nmol/L** | -0.086 | 0.918 | 0.690-1.219 |
|  | **50-<75 nmol/L** | -0.022 | 0.978 | 0.707-1.353 |
|  | **≥ 75 nmol/L*** | 0.368 | 1.445 | 1.002-2.091 |
|  | **Vitamin B12 (BL≥450 & FU≥450 pmol/L)** | Ref |  |  |
|  | **Vitamin B12 (BL<450 & FU≥450 pmol/L)*** | -0.114 | 0.892 | 0.733-1.086 |
|  | **Thyroid medication (yes)*** | 0.633 | 1.884 | 1.448-2.452 |
|  | **Season (Summer)** | Ref |  |  |
|  | **Season (Winter)** | -0.110 | 0.896 | 0.739-1.086 |
|  | **BMI kg/m^2^ (Normal weight)** | Ref |  |  |
|  | **Overweight*** | -0.743 | 0.476 | 0.373-0.606 |
|  | **Obese** | -0.016 | 0.984 | 0.738-1.313 |
| **CRP mg/L**  **R^2^=0.078**  **P<0.001** | **Age*** | 0.016 | 1.016 | 1.013-1.019 |
|  | **Gender (Male)*** | -0.189 | 0.828 | 0.761-0.901 |
|  | **BMI kg/m^2^ (Normal weight)** | Ref |  |  |
|  | **Overweight*** | -0.943 | 0.389 | 0.343-0.442 |
|  | **Obese*** | -0.272 | 0.762 | 0.637-0.912 |
|  | **25(OH)D improve (No improve)** | Ref |  |  |
|  | **<25 nmol/L** | 0.002 | 1.002 | 0.870-1.153 |
|  | **25-<50 nmol/L** | -0.025 | 0.976 | 0.854-1.115 |
|  | **50-<75 nmol/L*** | 0.183 | 1.201 | 1.042-1.384 |
|  | **≥ 75 nmol/L*** | 0.628 | 1.874 | 1.573-2.233 |
|  | **Vitamin B12 (BL≥450 & FU≥450 pmol/L)** | Ref |  |  |
|  | **Vitamin B12 (BL<450 & FU≥450 pmol/L)** | -0.071 | 0.931 | 0.852-1.017 |

^*^Binary Logistic Regression, TSH=Thyroid Stimulating Hormone, anti-TPO= anti-thyroid peroxidase antibody, anti-TG= anti-thyroglobulin, FT3=Free triiodothyronine, FT4=Free Thyroxine, CRP=C-reactive protein
